# Water-Tight Arthrotomy Joint Closure of Modified Intervastus Approach in Total Knee Arthroplasty

**DOI:** 10.3390/jcm12123985

**Published:** 2023-06-12

**Authors:** Muthana M. Sartawi, James M. Kohlmann, Karam R. Abdelsamie, Hafizur Rahman

**Affiliations:** 1 Department of Orthopaedics, Sarah Bush Lincoln Health Center, Mattoon, IL 61938, USA; 2 Department of Orthopaedics, King’s College Hospital London, Dubai 14143, United Arab Emirates; 3 Department of Orthopaedics, Minia University, Minia 61519, Egypt; 4 School of Podiatric Medicine, University of Texas Rio Grande Valley, Harlingen, TX 78550, USA

**Keywords:** total knee arthroplasty, joint closure technique, water-tight joint closure

## Abstract

Background: The joint closure technique used for total knee arthroplasty cases can have an impact on outcomes, especially when considering accelerated rehabilitation programs that follow surgery. In this study, we describe the details of the technical steps involved in performing the water-tight arthrotomy joint closure technique that we developed and use. Methods: A total of 536 patients (average age: 62 years, average body mass index: 34 kg/m^2^) with primary osteoarthritis of the knee underwent total knee arthroplasty using the modified intervastus approach between 2019 and 2021. We used the water-tight arthrotomy joint closure technique to close the knee arthrotomy incision. Any infections and complications, as well as the duration of surgery and cost related to this wound closure technique, are also reported. Results: Few complications were noted with this closure technique. When we first started using it, there was one case of drainage through the proximal capsular repair which required a return to the operating room 5 days postoperatively for an irrigation and debridement. We also had two cases of superficial skin necrosis along a small part of the incision line which were observed on a weekly basis and which healed uneventfully with application of betadine once daily on the necrotic area. The average time for performing wound closure after total knee arthroplasty was 45 min. Conclusion: We conclude that the water-tight closure approach can achieve very durable, water-tight capsule repairs and results in a decrease in postoperative wound drainage.

## 1. Introduction

Total knee arthroplasty (TKA) is one of the most common orthopedic surgical procedures, with more than 600,000 TKAs performed annually in the United States at present, and that number is expected to reach 3.48 million annually by 2030 [1,2]. Studies have reported significant improvement in functional outcomes after TKA, especially with the improved products and surgical techniques developed in recent years [3,4,5]. However, a considerable number of patients are not satisfied with the outcomes following TKA [6]. The joint closure technique is an important part of the TKA procedure. An effective joint closure can prevent infection and improve patient satisfaction with surgical outcomes [7].

Various knee arthrotomy closure techniques have been used for several years and include simple interrupted stitches, interrupted figure of eight stitches, a combination of simple and figure of eight stitches, and, least commonly, a running suture technique. In recent years, some surgeons have adopted closure with the recently developed barbed suture technique [7,8]. The barbed suture technique has been used in orthopedic and other surgeries and is characterized by knotless fixation [8]. The technique has been compared to interrupted stitches and was noted to have better waterproofing, higher cyclical tension, reduced surgical time, cost savings, and better cosmetic effects [7,8]. However, some studies have reported a higher frequency of arthrotomy failures when the barbed suture technique was used for arthrotomy closure after TKA [9]. Wright et al. also reported suture breakages when using barbed sutures for wound closures [10]. Studies have also documented an increased rate of superficial infections for barbed suture closures [11,12].

We developed a novel suture technique, which we named water-tight arthrotomy joint closure. The closure technique was developed to address demands regarding arthrotomy closure by patients who are aggressively pursuing an accelerated rehabilitation protocol after total knee arthroplasty. The technique permits accelerated rehab without the development of subcutaneous seromas, which can lead to wound drainage and ultimately infection. Additionally, increasing the use of fairly aggressive postoperative anticoagulation therapy contributes to the development of postoperative hemarthrosis and subsequent subcutaneous seromas/hematomas and wound drainage. In this paper, we describe the details of the technical steps involved in performing the water-tight closure technique to illustrate its ease of use. We also report the infections, complications, duration of surgery, and cost related to this wound closure technique. This paper presents an innovative arthrotomy closure technique that aims to preclude or markedly minimize the leakage of joint fluid and hematoma across the capsule suture line into the subcutaneous space. We hypothesize that by preventing the passage of joint fluid into the subcutaneous space, the incidence of wound drainage and associated complications can be substantially reduced. These findings bear clinical significance in the context of understanding the impact of wound closure techniques on postoperative wound complications. 

## 2. Materials and Methods

### 2.1. Patients

A total of 536 patients (average age: 62 years, average body mass index: 34 kg/m^2^) with primary osteoarthritis of the knee underwent TKA using the modified intervastus (MIV) approach between January 2019 and December 2021. In that time frame, patients who underwent revision total knee replacements were excluded from the study. The details of the MIV approach have been described previously [2,13,14]. We used the arthrotomy closure technique to close the knee arthrotomy incision. All surgeries were performed by an experienced, fellowship-trained orthopedic surgeon. Two well experienced surgeons performed the closures using the water-tight arthrotomy joint closure technique. 

### 2.2. Water-Tight Arthrotomy Joint Closure Technique

**Figure 1:**
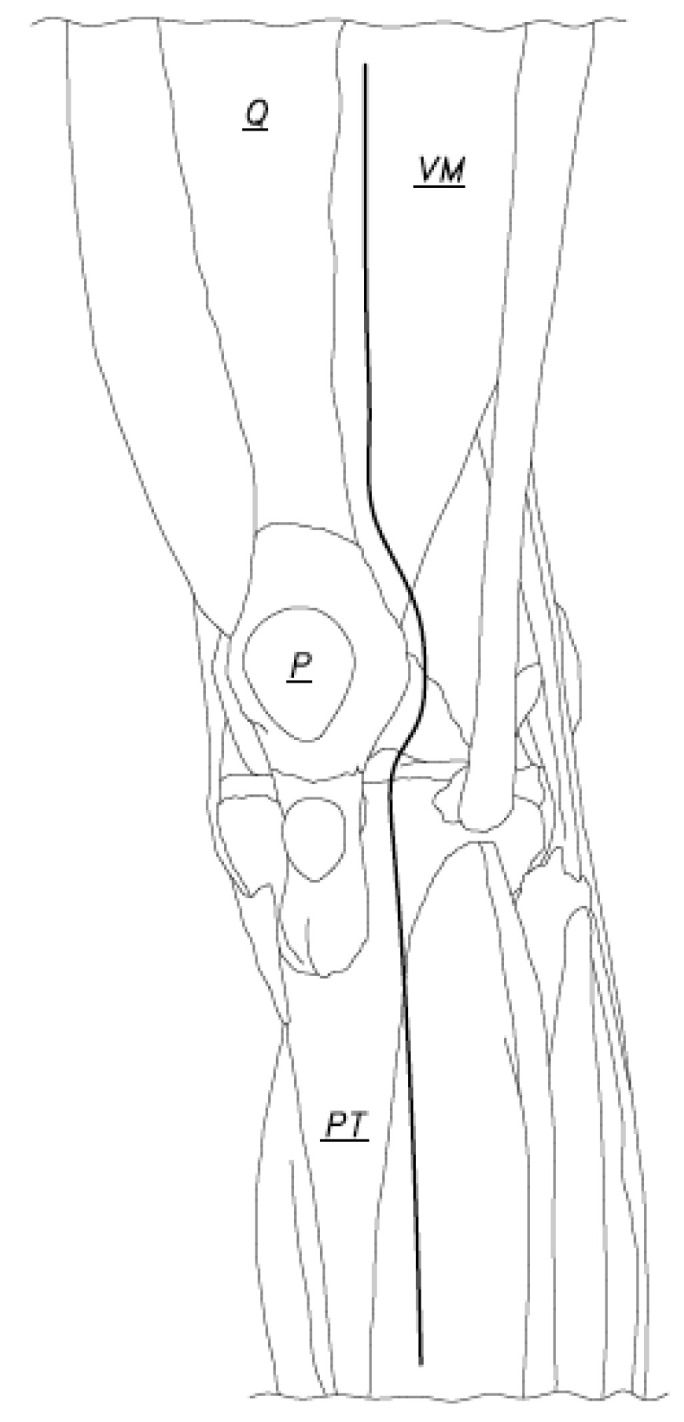
The bold line indicates the arthrotomy incision (Q: Quadriceps tendon, VM: Vastus Medialis, P: Patella, PT: Patellar tendon).

**Figure 2:**
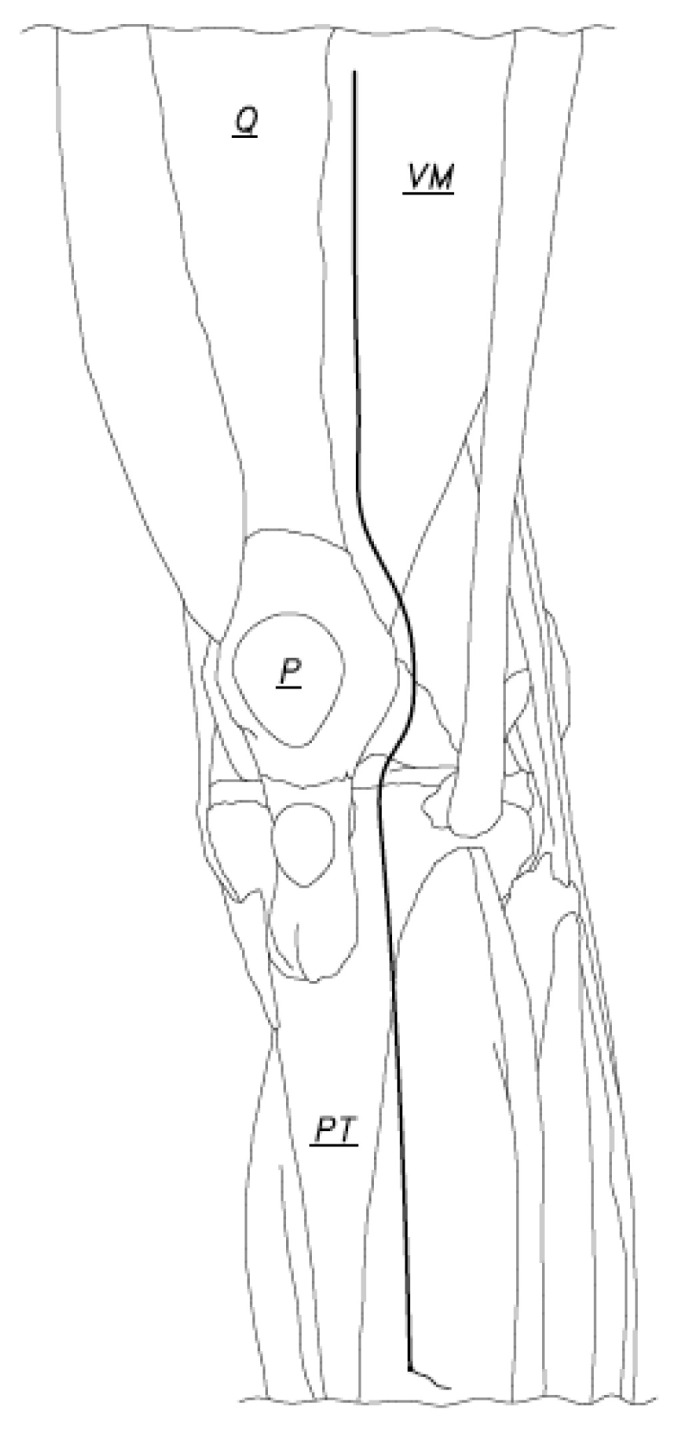
Closure began at the caudal end of the arthrotomy incision and was done with the knee in 90 degrees of flexion. The suture was passed at the caudal end of the arthrotomy incision and was tied using two or three knots (Q: Quadriceps tendon, VM: Vastus Medialis, P: Patella, PT: Patellar tendon).

**Figure 3:**
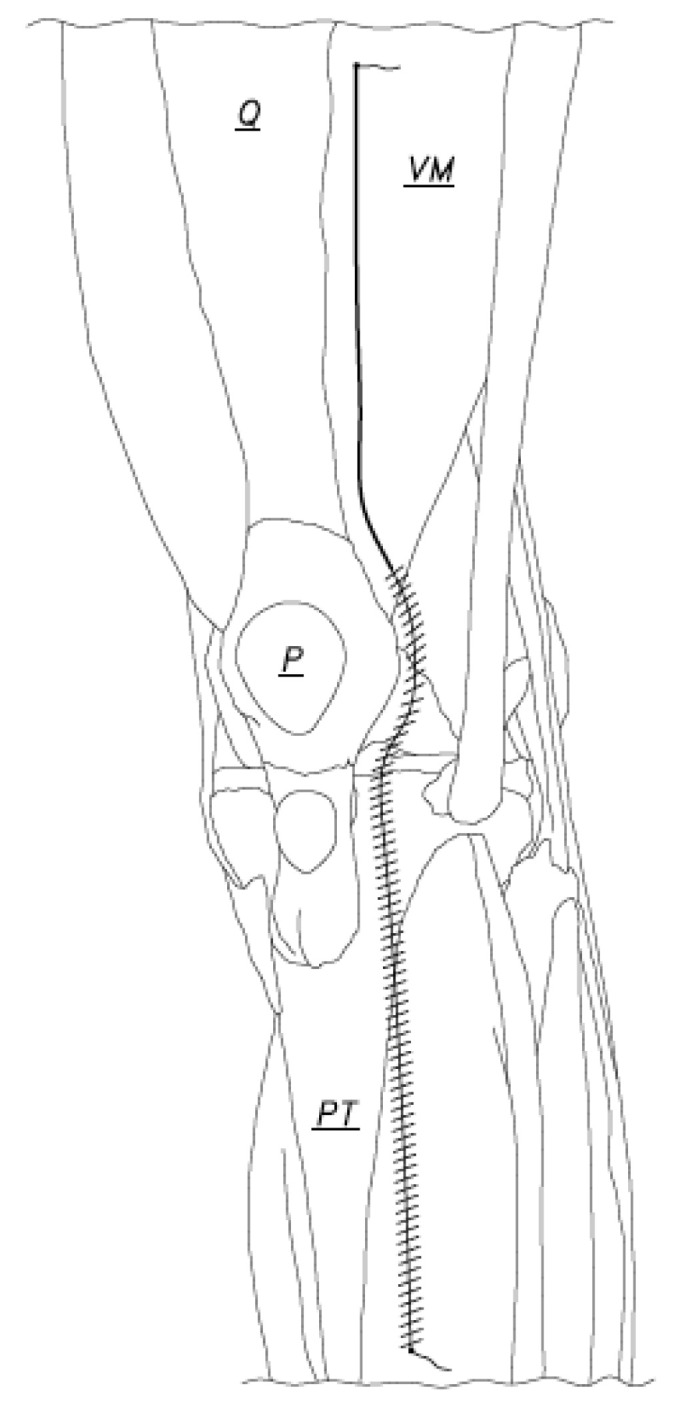
The arthrotomy was closed using a running stitch from the caudal end of the arthrotomy moving cephalad (distal hashed line). When the capsule repair to or just beyond the superior pole of the patella has been accomplished, the suture was laid to rest to begin repair of the capsule of the knee at the cephalad end of the arthrotomy (Q: Quadriceps tendon, VM: Vastus Medialis, P: Patella, PT: Patellar tendon).

Arthrotomy closure was accomplished using two number one vicryl sutures (our choice), but the suture of choice was good (Figure 1). Closure began at the caudal end of the arthrotomy incision and was done with the knee in 90 degrees of flexion (Figure 2). The suture was passed at the caudal end of the arthrotomy incision and was tied using two or three knots. The one-inch-long tail left after the suture was tied was not cut short (Figure 2) and was later used. The arthrotomy was then closed using a running stitch anchored at the just created caudal anchor site and passed through the capsule edges, which were made parallel to each other and were no more than 4 or 5 mm apart. When the capsule medial to the patella was reached, the repair continued as just described, but less flexion of the knee (50 degrees flexion, for instance) made repair of the capsule in this region much easier to accomplish. When the capsule repair to or just beyond the superior pole of the patella was accomplished, the suture was laid to rest for a moment and a second suture was used to begin the repair of the capsule of the knee at the cephalad end of the arthrotomy (Figure 3).

**Figure 4:**
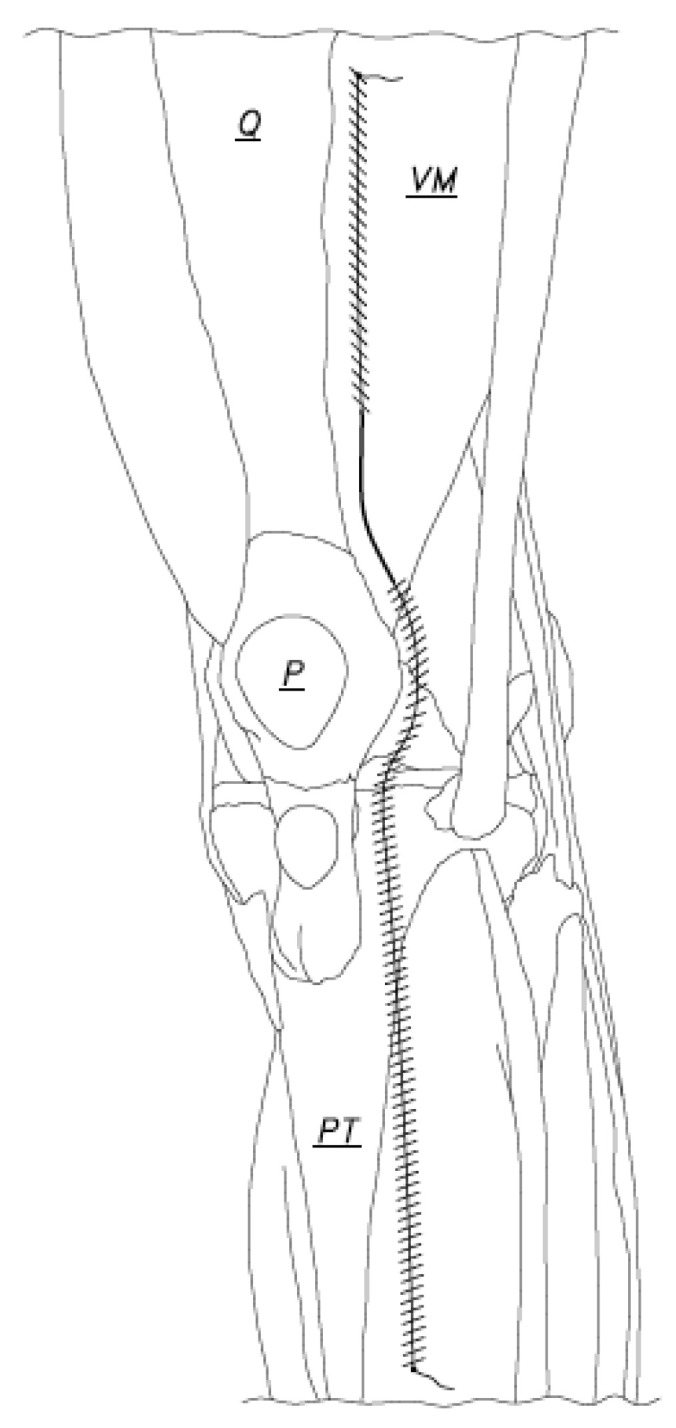
The second suture was passed through the cephalad end of the capsulotomy and tied leaving a 1 ½ inch suture tail intact to be used later. The proximal capsule was then sutured (proximal hashed line) (Q: Quadriceps tendon, VM: Vastus Medialis, P: Patella, PT: Patellar tendon).

**Figure 5:**
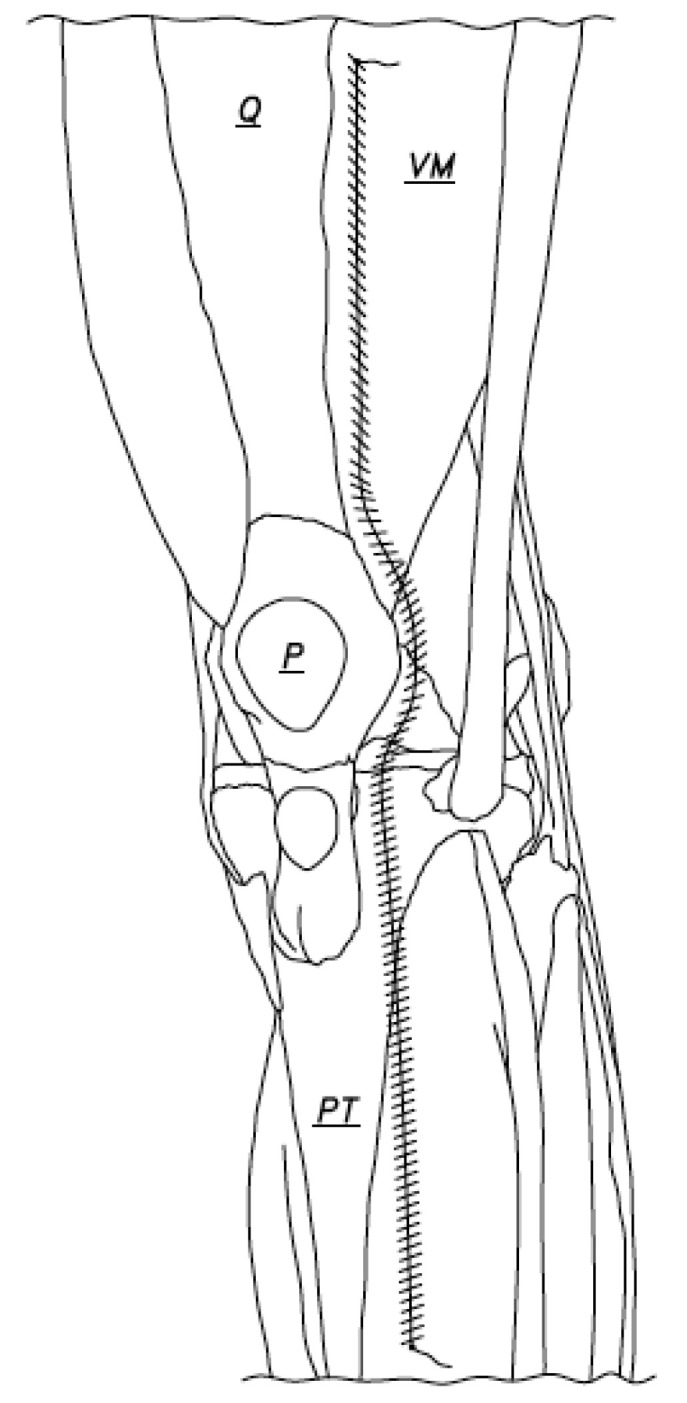
When the capsule had been closed completely, the sutures met in the middle of the arthrotomy (Q: Quadriceps tendon, VM: Vastus Medialis, P: Patella, PT: Patellar tendon).

**Figure 6:**
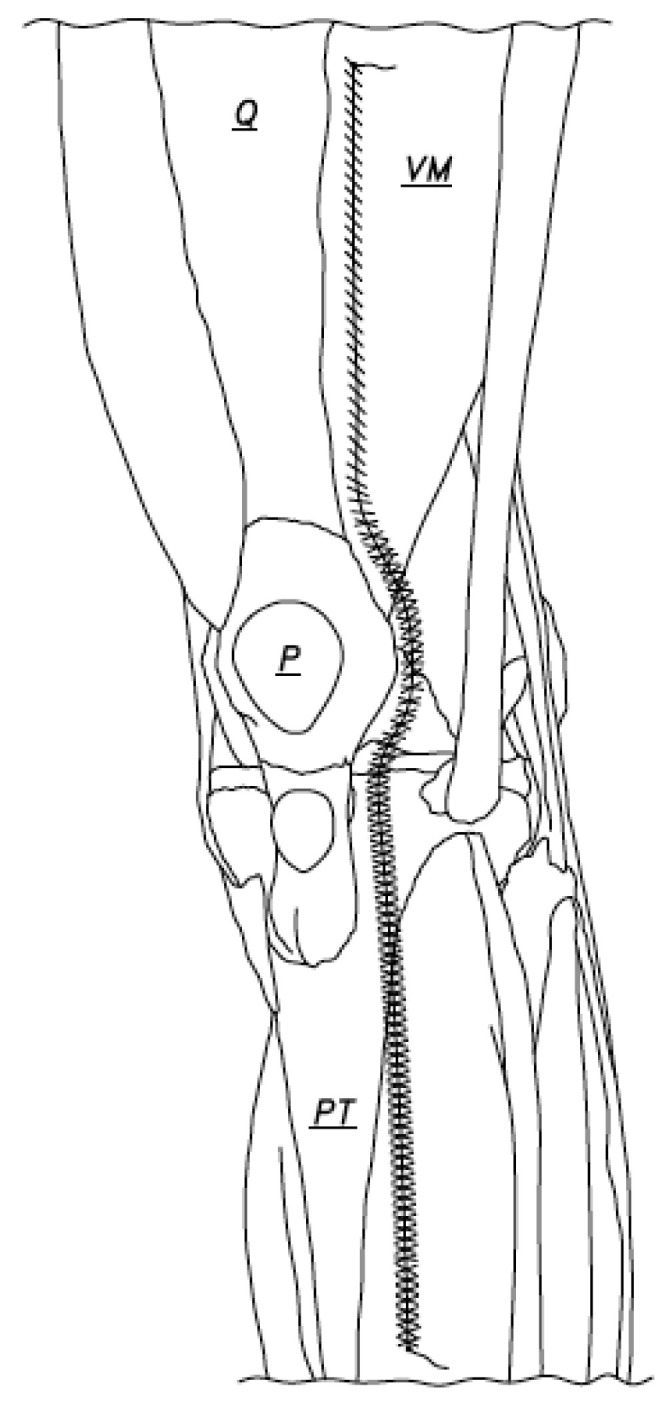
The repair continued as the suture anchored at the cephalad position was passed through the already repaired capsule (distal double hashed line) all the way to the caudal edge of the arthrotomy. This suture was then tied to the tail of the anchor stitch at the distal arthrotomy edge, at which time the tails of the tied suture could be cut (Q: Quadriceps tendon, VM: Vastus Medialis, P: Patella, PT: Patellar tendon).

The knee should be in less than 90 degrees flexion to facilitate passing the anchor stitch and to place a few more passes to repair the capsule. The anchor stitch was tied leaving a 1 ½ inch tail to be used later (Figure 3). The knee should be at 90 degrees flexion for most of the rest of the repair. The capsule was then repaired using a running suture technique, as was described for repairing the distal half of the capsule (Figure 4). The capsule was closed completely when the sutures met at the superior pole of the patella (Figure 5). The capsule repair continued by maintaining the 90-degree flexion position of the knee, adjusting the flexion as necessary as the suture anchored at the cephalad position was passed through the already repaired capsule medial to the patella and then all the way to the caudal edge of the arthrotomy. This suture was then tied to the tail of the anchor stitch at the distal arthrotomy edge and the tied suture was finished by cutting the tails (Figure 6). 

The 90-degree knee flexion position was maintained as the Vastus Medialis Oblique (VMO) fascia was repaired up to the quadriceps tendon edge by running suture number 1, which had been temporarily left untouched at the superior pole of the patella (Figure 6). The suture passes were about 4 mm apart and were parallel to each other. When the proximal anchor suture tail was reached and the fascia was anatomically reapproximated, the suture was tied to the tail that was left intact after the proximal anchor stitch had been tied. The suture was then cut.

**Figure 7:**
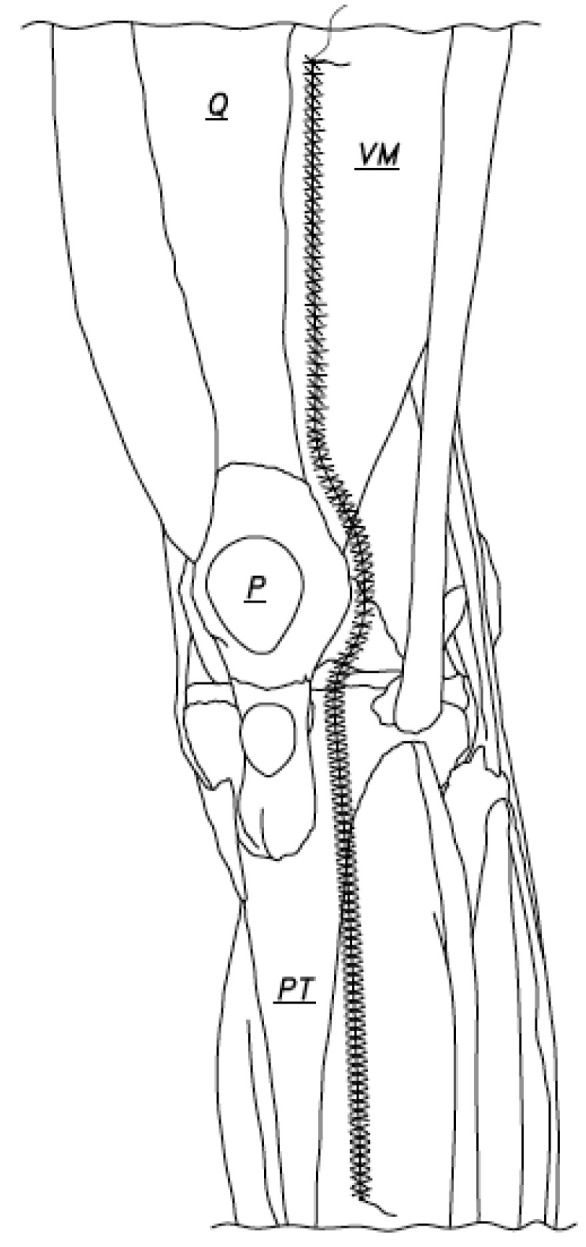
The Vastus Medialis Oblique (VMO) fascia was repaired up to the quadriceps tendon edge by running the number 1 suture anchored at the superior pole of the patella (proximal double hashed line). When the proximal anchor suture tail was reached and the fascia was anatomically reapproximated, the suture was tied to the tail that was left intact after the proximal anchor stitch had been tied (Q: Quadriceps tendon, VM: Vastus Medialis, P: Patella, PT: Patellar tendon).

If the arthrotomy was made through the quadriceps tendon, as was commonly done by many surgeons, the quadriceps tendon repair already carried out by the proximally anchored suture was reinforced by the passage of the distally anchored suture and in a running fashion, as described above for repair of the VMO fascia (Figure 7). This repair, when properly done, provides a water-tight closure which can be checked by injecting saline or other fluid and checking for leaks. Areas found to leak can be reinforced with an additional suture. 

In our TKA procedure, a tourniquet was not utilized. Patients received 1 g of tranexamic acid, administered just prior to incision and closure. All arthrotomies and skin incisions were closed over small hemovac drains, which were removed on postoperative day 1 or 2, depending on the amount of drainage. Low-dose aspirin was used postoperatively for thromboembolism prophylaxis for 28 days.

### 2.3. Outcome Assessment

Any infections or complications related to wound closure were recorded. Included here were both superficial and deep infections, cellulitis, and any wound dehiscence, be it superficial or deep. The observed superficial wound dehiscence was mainly an opening of the skin only. Wound dehiscence could include both skin and capsule. Finally, the skin could remain intact while the capsule repair failed or dehisced. These wound dehiscence problems could present at any time in the early postoperative period. 

We recorded the duration of surgery as the time from skin incision to the time of complete skin incision closure, including the drying time for the skin adhesive. The time was recorded by a certified nurse during wound closures. 

We also reported the cost related to the water-tight wound closure technique. Increased durations of surgery increase cost and, theoretically, complications, such as postoperative infection.

## 3. Results

### 3.1. Infections and Complications 

Few complications were observed with this closure technique. When we first started using it, there was one case of drainage through the proximal capsular repair which required a return to the operating room 5 days postoperatively for an irrigation and debridement. The intraoperative cultures were negative, and the patient had an uneventful recovery. We also had two cases of superficial skin necrosis along a small part of the incision line, both of which were observed on a weekly basis, and which healed uneventfully with application of betadine once daily on the necrotic area. No patients had a postoperative infection at more than 3 months postoperative. 

### 3.2. Operative Time

The average time for performing wound closure after TKA was 45 ± 6 min. This closure time included closure of the capsule arthrotomy and skin. 

### 3.3. Cost

The cost of using this arthrotomy closure instead of, say, a faster one can be thought of as having two parts. The first part is the cost of any additional operating time and the corresponding increasing operating room time expense. The second part is any increased cost of the materials used to perform the closure. The water-tight arthrotomy closure described in this paper could add 10 min to the capsule closure time, which adds approximately $400, assuming that the operating room cost is $40.00 per minute [15]. Capsule closure is easily accomplished using two number 1 vicryl sutures at a cost of $1.80 per suture, or a total of $3.60. A subcuticular 3-0 vicryl skin closure finished with Perineo tape and Dermabond adds at least 20 min of operative time at a cost of $800. The material cost for this closure is therefore $3.46 for the suture and $20 for the Perineo and Dermabond. 

## 4. Discussion

The joint closure technique chosen by surgeons for total knee arthroplasty patients should be very durable, water-tight, and preferably devoid of suture material or staples that penetrate the skin from the outside to at least the subepidermal layer. Skin staples have been well documented in the literature to become colonized, both superficially and deep within the epidermis surface, and this could have implications for postoperative wound infection. The water-tight arthrotomy closure technique introduced in this paper presents an alternative closure method worth considering because it meets the desirable closure goals described in this paper. We also report operative time, any infections or complications related to would closure, and any increased cost associated with its use.

Recently, the barbed suture technique has gained popularity in capsular closure compared to interrupted sutures. Although the barbed suture technique can achieve better water-tight closure, cost saving and reduced surgical time [7], there are studies that have reported a higher frequency of arthrotomy failure after TKA [9].

**Figure 8:**
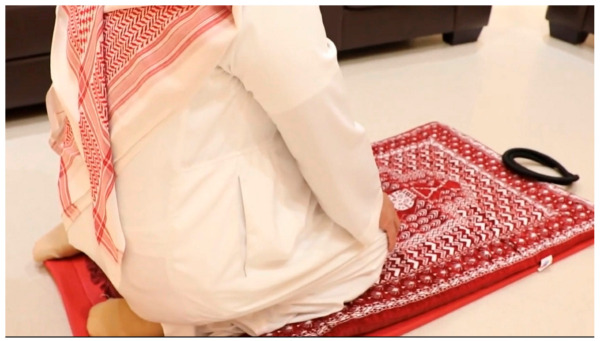
A patient successfully kneeling for prayer on the operated right knee, a mere four hours post TKA. This image exemplifies the early postoperative functionally achieved through the water-tight closure technique.

Capsular repair performed using this water-tight closure technique provided a durable and sturdy repair that remained intact, even when early postoperative demands were placed on the repaired capsule as patients progress through a rapid recovery protocol. Furthermore, the technique allowed patients to resume activities such as kneeling for prayer at a very early stage of recovery without compromising the repair’s integrity (Figure 8). These favorable outcomes are attributed to the enhanced strength and resilience afforded by the water-tight closure technique, proving it to be a sturdy and effective approach in capsular repair during TKA. An exceptionally low incidence of subcutaneous seromas in the postoperative period was observed, which we believe was due to the fairly water-tight closure of the capsule. A significant amount of subcutaneous fluid would precede wound drainage. Only one patient in this entire series experienced wound drainage in the early postoperative period, which was addressed surgically by exploring the capsule repair. Fluid was found to be leaking from the joint into the subcutaneous area through one small area of the capsule repair located at the cephalad end of the repaired capsule. The area was sutured, and the leak promptly stopped. The patient went on to heal uneventfully.

Accurately assessing or calculating the risk of developing capsular necrosis using this technique was, for us, difficult at best. Thus, we took measures to enhance repair precision while minimizing the potential for tissue strangulation. These measures included closing the wound while the knee was flexed at 90 degrees, which facilitated an accurate match-up of the capsule for repair and represented the capsule’s length under repair. Additionally, we maintained a small space between suture passes through the capsule to reduce the tension on the suture at each pass site. Our hypothesis was that multiple suture passes were stronger and posed a lower risk of tissue strangulation than fewer passes. During the repair process, we tensioned the suture just enough to nicely approximate the capsule being repaired, without applying aggressive tension. Placing the stitches close together brought the capsule together with less tension than spacing the stitch passes farther apart. We chose a repair technique that utilized two sets of running sutures, thereby requiring less tension. Finally, we gained experience with this technique and did not observe any of the capsule disruptions that would presumably result from capsule necrosis in this series of patients.

Accelerated recovery programs place significant demands on the knee in the very early postoperative period. Arthrotomy (capsule) dehiscence with intact skin closure is one problem that can result from aggressive knee motion exercises. Wound drainage in the early postoperative period is extremely undesirable. We strongly suspect that wounds that develop drainage do so because the knee joint seroma/hematoma fluid that is present in all patients to some degree in the early postoperative period moves through the repaired capsule to the subcutaneous tissue, where it accumulates until enough pressure is present to cause the fluid to pass through vulnerable areas in the skin suture line. In instances of capsular dehiscence where the skin remains repaired, drainage is all but guaranteed. We developed a capsule closure technique that addresses both the strength and reliability of the capsule repair and the problem of early postoperative wound drainage. Certainly, the elimination of wound drainage comforts surgeons and patients and is likely significantly associated with a decrease in postoperative infection rates. The closure we describe results in a very durable capsule repair that is water-tight and which, when combined with a subcuticular skin closure sealed with Perineo tape and dermabond, results, in our experience, in complete elimination of postoperative wound drainage [16]. For postoperative rehabilitation, patients started static and dynamic quadriceps exercises on the day of surgery, along with active knee range of motion exercises, which were continued daily under the supervision of an experienced physical therapist for two weeks. Physical sessions continued for an average of three sessions per week for three months. 

The average surgery durations for other wound closure techniques for TKA, such as staples and subcuticular suture, were found to be 94.1 min and 96.6 min, respectively [6]. In contrast, our water-tight arthrotomy repair with running subcuticular vicryl and Prineo tape adds 45 min to the total duration after the implant has been completely installed. If other techniques require 15 min of operative time to complete closure, then the duration of surgery using our wound closure technique adds 30 min to wound closure time. Decreasing the duration of surgery is considered a desirable practice, as noted in the literature [17]. Intraoperative measures/procedures that increase the duration of surgery but which eliminate postoperative wound problems like wound drainage, which is known to have an association with surgical site infections, have not been associated with an increase in postoperative complications of any kind in our practice. The practice of closing the skin with subcutaneous vicryl to meticulously reapproximate the skin edges and then finishing skin closure with Dermabond adds time, and this time is included in the 45 min of closure time mentioned above. Accurate information regarding the extent of additional protection from wound drainage provided by this type of skin closure should be pursued with a well-designed study dedicated to obtaining such knowledge. 

Any increased cost associated with the water-tight closure method we have described is primarily attributable to the increased time that the technique usually requires. If the technique does substantially achieve the closed TKA wound goals of substantial closed wound durability and elimination of wound drainage, even in patients participating in an accelerated recovery program, then the additional operative time, efforts, and associated costs are justified. Judging only by the materials used, the water-tight wound closure procedure described in this paper is economical when compared to other durable arthrotomy closures [18].

This water-tight closure technique has applications in total joint closures and in closures of any fascial tissues. We have employed its equivalent in fractures and other case types where applicable. The repairs are quite durable and certainly help reduce subcutaneous seromas and drainage. In the case of knee arthroplasty, our experience is that postoperative wound problems are very infrequently seen. We also did not observe any capsule repair failures in this series of patients. These findings have great value in clinical practice. The strengths of this study are its inclusion of all primary knee arthroplasty patients, the follow-up findings, and its attempt to link the quality of capsule repair to wound problems which are routinely encountered in the postoperative period. 

This study had several limitations. One such limitation was that our outcome measurements were limited to infections and complications, operative time, and cost. Our closure technique takes more time than closing the capsule with one running barbed suture and maybe more time than that required for multiple interrupted sutures. Furthermore, we did not statistically compare our outcomes with those reported using other existing suture techniques. Future studies could compare the water-tight closure technique described here with other closure techniques, utilizing outcome measures such as the Knee Society score, functional score, patient satisfaction visual analog scale, incision length, estimated blood loss, and knee range of motion. 

## 5. Conclusions

We conclude that the closure described herein results in a very durable, water-tight capsule repair that is related to a decrease in postoperative wound drainage. 

## 6. Patents

Muthana M. Sartawi is the owner of the patent “Modified Intervastus Arthrotomy Closure Method” (Patent Number: 11553910). 

## Data Availability

The datasets generated and used in the current study are available on reasonable request from the corresponding author.
